# When the print got small

**DOI:** 10.51866/mol.1137

**Published:** 2026-05-01

**Authors:** Mohamed-Syarif Mohamed-Yassin

**Affiliations:** 1 Department of Primary Care Medicine, Faculty of Medicine, Universiti Teknologi MARA, Sungai Buloh Campus, Sungai Buloh, Selangor, Malaysia.

**Keywords:** Ageing, Old age, Fitness to practise, Physician health, Reflections

There are certain milestones in life that may prompt us to pause and reflect.

The ‘critical incident’ for me occurred when I struggled to read the small print of a medication expiry date. This was frustrating, as I have always needed glasses for myopia, yet I also now experience difficulty reading up close. To think that I felt slightly offended when my optometrist wanted to test my near vision during a routine eye examination when I turned 40!

There are also other signs of ageing. The presence of grey hair has been a common occurrence for some time. Further, there are more subtle signs – creaking joints and my lower back becoming stiff after prolonged periods of sitting.

Many friends are increasingly being diagnosed with heart attacks, needing cardiac stenting or bypass and requiring long-term medication for high blood pressure or diabetes. Another sign is that my children are also becoming older. The music video for ‘Saat Kau Telah Mengerti’ by Indonesian singer Virgoun has begun to resonate with me profoundly.^1^ It depicts the passage of time and the changing of roles, themes that carry greater significance for me now.

Within my professional environment, many senior colleagues have retired, and others will do so soon. I have approximately 15 years of service remaining before reaching my formal retirement age. This period seems long until I realised that I have already spent a decade in academia. In Malaysia, there is no statutory maximum age for the practice of medicine. However, the Malaysian Medical Council offers guidance for those who wish to continue practising beyond the age of 70.^2^ This also somehow reframes the concept of time, as we do not merely count the years left but instead consider how to remain fit to practise within them.

However, ageing is not entirely characterised by decline. The book The Changing Mind by Daniel Levitin comforts me with a reminder that our lifelong expertise, the ‘crystalised’ wisdom we have spent decades building, the ‘gut’ instinct we use during complex consultations, only continues to deepen.^3^

Levitin also argues that our brains stay sharp when we tackle unpredictable environments, not just repetitive ones. Hence, although it is more convenient to run on a treadmill at home, I have forced myself to run outdoors at least once a week. Honestly, this requires more time and effort. Feeling the uneven pavement under my feet and the humidity against my skin is a stark contrast to the comfort of my home. However, that is precisely the point. Every stumble over a loose stone and the mugginess of the morning heat represent an active choice to stay adaptable. It is a small and sweaty way of reminding myself that even after decades in medicine, I remain a work in progress.

As family medicine specialists, we are adept at finding solutions to our patients’ problems. Hence, when the print gets small, we do not stop reading – we simply magnify it.
